# Cognitive control: exploring the causal role of the rTPJ in empathy for pain mediated by contextual information

**DOI:** 10.1093/scan/nsae057

**Published:** 2024-09-06

**Authors:** Helena Hartmann, Egle M Orlando, Karina Borja, Christian Keysers, Valeria Gazzola

**Affiliations:** Social Brain Lab, Netherlands Institute for Neuroscience, Royal Netherlands Academy of Art and Sciences, Amsterdam 1105 BA, The Netherlands; Clinical Neurosciences, Department of Neurology and Center for Translational and Behavioral Neurosciences, University Hospital Essen, Essen 45147, Germany; Social Brain Lab, Netherlands Institute for Neuroscience, Royal Netherlands Academy of Art and Sciences, Amsterdam 1105 BA, The Netherlands; Department of General Psychology, University of Padua, Padua 35131, Italy; Social Brain Lab, Netherlands Institute for Neuroscience, Royal Netherlands Academy of Art and Sciences, Amsterdam 1105 BA, The Netherlands; Social Brain Lab, Netherlands Institute for Neuroscience, Royal Netherlands Academy of Art and Sciences, Amsterdam 1105 BA, The Netherlands; Brain and Cognition, Department of Psychology, University of Amsterdam, Amsterdam 1018 WT, The Netherlands; Social Brain Lab, Netherlands Institute for Neuroscience, Royal Netherlands Academy of Art and Sciences, Amsterdam 1105 BA, The Netherlands; Brain and Cognition, Department of Psychology, University of Amsterdam, Amsterdam 1018 WT, The Netherlands

**Keywords:** rTMS, rTPJ, contextual information, mentalizing processes, empathy for pain

## Abstract

Empathy determines our emotional and social lives. Research has recognized the role of the right temporoparietal junction (rTPJ) in social cognition; however, there is less direct causal evidence for its involvement in empathic responses to pain, which is typically attributed to simulation mechanisms. Given the rTPJ’s role in processing false beliefs and contextual information during social scenarios, we hypothesized that empathic responses to another person’s pain depend on the rTPJ if participants are given information about people’s intentions, engaging mentalizing mechanisms alongside simulative ones. Participants viewed videos of an actress freely showing or suppressing pain caused by an electric shock while receiving 6 Hz repetitive transcranial magnetic stimulation (rTMS) over the rTPJ or sham vertex stimulation. Active rTMS had no significant effect on participants’ ratings depending on the pain expression, although participants rated the actress’s pain as lower during rTPJ perturbation. In contrast, rTMS accelerated response times for providing ratings during pain suppression. We also found that participants perceived the actress’s pain as more intense when they knew she would suppress it rather than show it. These results suggest an involvement of the rTPJ in attributing pain to others and provide new insights into people’s behavior in judging others’ pain when it is concealed.

## Introduction

Human empathy, which can be understood as our ability to understand and share the affective states of others, is crucial to our everyday social interactions and is a fundamental component of social intelligence (Singer et al. 2004). As social beings, watching others suffer in reality, or even just observing them in the media, resonates strongly within us.

Typically, research distinguishes between two complementary parts of empathy: cognitive empathy, which is the ability to cognitively infer the affective state of another person (Van Overwalle and Baetens 2009), and emotional empathy, which is an affective state resulting from a partial and experiential sharing of another person’s affective state (Bernhardt and Singer 2012). Cognitive empathy and top-down regulation processes of affective states are also strongly related to self–other distinction (SOD), the ability to differentiate self- and other-related affective states (Shamay-Tsoory 2011, Bukowski et al. 2020).

Cognitive empathy and emotional empathy appear to operate, at least in part, independently on the neural level. Whereas cognitive empathy often recruits the so-called mentalizing network (e.g. the ventromedial prefrontal cortex and temporoparietal junction; TPJ), emotional empathy recruits networks related to emotion recognition and contagion (e.g. the inferior frontal gyrus and intraparietal lobule; Shamay-Tsoory 2011). Although the overall empathic response may be based on the complex interaction of both processes (Keysers and Gazzola 2007), depending on the social context, it is believed that emotional and cognitive empathy represent two pathways to understanding others (Zaki and Ochsner 2012, Spunt and Lieberman 2013, Zaki 2014).

A key hub of the mentalizing network and cognitive empathy is the right temporoparietal junction (rTPJ), whose important role in social cognition is widely recognized through an abundance of research. Indeed, the rTPJ is recruited during SOD, imitation control, and agency processing (Ruby and Decety 2001, Farrer and Frith 2002, Saxe and Wexler 2005, Saxe and Powell 2006, Brass et al. 2009). Furthermore, the rTPJ activates during the adoption of the perspectives and beliefs of others (Van Overwalle 2009, Schurz et al. 2014), and when recognizing false beliefs or encoding that someone’s mental state may differ from visible evidence (Özdem et al. 2019). While the close connection between rTPJ activity and social cognition is well established in correlational studies, its causal role in the recognition or interpretation of someone’s emotions is still unclear (Paracampo et al. 2018).

The investigation of people’s ability to evaluate the emotions of others has often used pain as a model, potentially due to the robustness of pain in inducing empathic responses in an observer. First-hand pain is, in fact, highly motivational and can induce adaptive avoidance (Price 2000) and warning behaviors among conspecifics (Craig 2004), whereas third-person pain motivates helping behaviors (Hein et al. 2010, Gallo et al. 2018). Moreover, research on empathy for pain has provided ample evidence of the neural circuits involved in this experience (e.g. somatosensory cortices 1 and 2, anterior cingulate cortex, midcingulate cortex, and anterior insula; Bernhardt and Singer 2012). When people are asked to assess others’ pain from facial expressions, they rely on regions associated with emotional empathy and simulation (Soyman et al. 2022). There is only some causal evidence for the rTPJ’s involvement in processes of attributing pain to others (Coll et al. 2017, Miller et al. 2020). Nevertheless, to the best of our knowledge, no prior study has introduced contextual information as a variable, or investigated whether the involvement of the TPJ in the process of attributing pain to others is dependent on such information.

It was recently proposed that, in some situations, simulation and mentalizing networks may work together, opening up the perspective that these processes may work synchronously rather than in isolation, and that the attribution of others’ pain may rely on both networks rather than on simulation processes alone (Keysers and Gazzola 2007). In line with this integrative view and the evidence of the causal involvement of the rTPJ in holding false beliefs, inferring someone’s level of pain from their facial expression might rely on the rTPJ if the inferring person is aware that the other person is trying to hide their pain. In this type of situation, empathic inference about others’ pain may have to leverage not only typical simulative processes but also mentalizing processes.

Other evidence partially supporting this notion is the role the TPJ plays in encoding contextual information in relation to social scenarios (Gu et al. 2019). In particular, research has proposed that the TPJ may be necessary in mediating the social framing effect or when a change in the description of a social dilemma (or a specific social component of this dilemma) significantly modulates a decision-maker’s preference toward different options (Liu et al. 2020). In line with this, it is possible that the TPJ may also be sensitive to contextual information given around an emotion evaluation as part of an empathic experience. The contextual updating hypothesis (Geng and Vossel 2013) suggests that the TPJ is essential for updating internal models of the internal or external context by integrating new information from the stimulus. TPJ recruitment in this process would be increased when the new information does not match the expectations of one’s internal model. The TPJ may thus be necessary to update the expectations of the internal model when contextual information is provided about the intentions of a person to suppress or show pain.

To answer this question, we developed a task in which an actress is shown receiving painful stimuli under two conditions: either freely expressing her pain or suppressing it. Before observing the videos and rating the actress’s pain levels, participants were informed that the actress had been instructed to either express or suppress her pain (video stimuli labeled as pain being shown or suppressed, respectively). Along with the ratings, response times (RTs) were also assessed to investigate possible variations in inferential demand under the two conditions, especially considering that numerous studies have shown increased activation in the rTPJ as inferential demands increase (Vistoli et al. 2016, Henry et al. 2021). To investigate the causal role of the rTPJ, participants received 6 Hz repetitive transcranial magnetic stimulation (rTMS) over the rTPJ or sham stimulation over the vertex during the task.

Based on previous findings, we hypothesized that: (i) participants would perceive more pain when the actress expresses pain compared to when she suppresses it (main effect of Video on ratings); (ii) participants would perceive more pain when they believe that the actress is suppressing her pain rather than expressing it (main effect of Label on ratings); and (iii) rTMS on the rTPJ would disrupt the effect of Label on ratings or RTs (TMS × Label interaction). Given the contextual updating hypothesis, rTMS might also disrupt the correct decoding of facial expressions of pain and their relationship to contextual information, especially if the information provided does not align with the actual emotions observed (TMS × Video × Label interaction).

## Materials and methods

### Participants

Healthy participants between 18 and 40 years of age who fulfilled the safety criteria for TMS were invited to participate in the present study. Participants were screened for these criteria and only invited to the study if they passed the safety screening for TMS collected twice, during recruitment and at the experimental session. Fourteen participants [10 women, 4 men, mean age (s.d.) from 9/13 participants = 26.00 (2.76) years, age range = 24–34 years] participated in the present study. All participants provided written consent, and the study was accepted by the Medisch Ethische Toetsingscommissie of the Amsterdam Medical Center (application number 2019_025) prior to the start of the study. The whole experiment lasted 3 h for each participant, and the participants received 10 Euros per hour for their participation.

### Procedure

First, we measured participants’ resting motor threshold (rMT) over the right hemisphere. To do so, we measured the distance from inion to nasion to identify the center of the head by connecting the two at the halfway mark. This line was crossed with the ear-to-ear line, and the center of the brain was set as the location where the two lines met (corresponding to Cz as measured by the international 10–20 system). We marked the approximate region of the right motor cortex as the right front quadrant. We used a MagStim Rapid^2^ stimulator and a Figure-of-8 coil (D70 Alpha coil; Magstim Co. Ltd, diameter 70 mm) for all stimulations during the assessment of the rMT and the task. Then, we applied single stimulations of gradually increasing intensity (starting with 45% of the stimulator’s output intensity and increasing in steps of 5%, single pulses at least 6 s apart). The intersection of the coil was placed tangentially to the participant’s scalp over the right front quadrant, with the handle pointing backward, and laterally at a 45° angle away from the midline. Using three electromyography (EMG) Ag–AgCl surface electrodes (Ø35 mm) on the left hand (one for the muscle, one for the index finger knuckle, and one for the ground on the inner wrist), following the standard belly-tendon EMG montage, we recorded the muscle response of the first dorsal interosseous muscle to TMS. The signal was visualized with Acquisition Software (EMGworks), and through the combination of this information and participants’ reports of muscle twitching (participants indicating once they felt a sensation in their left hand or index finger), we determined the motor hotspot and gradually decreased the intensity again to find the lowest stimulation intensity needed to produce motor-evoked potentials (MEPs) of 0.05 mV peak-to-peak (Rossini et al. 2015). We then tested this location using 10 pulses at the specified intensity by checking whether at least 5 of the 10 pulses resulted in MEPs around 0.05 mV (mean rMT/s.d. = 51.71%/3.29) and then set the stimulation intensity for the subsequent task at 90% of this value (mean stimulation intensity/s.d. = 46.00%/3.56). Next, we marked the vertex (location Cz) on the scalp. If the participants had a stimulation intensity >60% of the stimulator’s output intensity or reported the stimulation to be too unpleasant to handle in the first trials, they were not further tested due to the unpleasantness of the stimulation. This approach was chosen based on prior experience of the authors and colleagues with TMS expertise. The right TPJ was set on the following coordinates (*x* = 51, *y* = −54, *z* = 21) of Talairach space [Montreal Neurological Institute space (51, −56, 20)] that have been identified on the basis of neuroimaging studies exploring areas related to mentalizing (Van Overwalle and Baetens 2009, Mar 2011, Bzdok et al. 2012).

Prior to the study, video stimuli were generated displaying a woman receiving real but tolerable shocks of varying intensity on her right hand and eliciting facial expressions of pain in line with the stimulation, ranging from mildly to annoyingly painful stimulation. During the prerecorded videos, the woman was instructed to either freely show or suppress her facial expressions of pain in response to the shocks. Each video started with a neutral expression; following the delivery of the shock, it led to a gradual change in facial expression until 1 s after the video started. For the last second, the facial expression gradually changed back to neutral. The final choice of video stimuli used in the present study was based on two previous pilots, with 20 subjects each, designed specifically to select from all the videos generated those that elicited the greatest empathic accuracy between the pain ratings of both the actress and the participants (i.e. the concordance between the ratings). This resulted in 104 independent show and 72 independent suppress videos showing the actress receiving electrical stimulation of varying intensity per block, which were used for the final task. These stimuli were fully randomized across participants and every video was thus shown for minimum two and maximum three times. Videos were generated counterbalancing for pain intensity and did not differ in empathic accuracy. The video duration was 2 s, following an indefinite rating period and 7 s of inter-trial-interval (ITI) (including a 1-s warning screen) before the subsequent video/stimulation.

**Figure 1.**
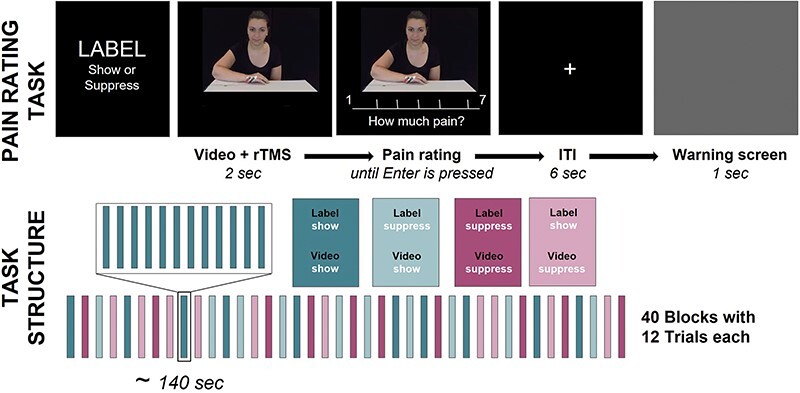
Participants saw videos of a female receiving electrical stimulation in varying intensities on her hand and then rated the pain they thought the woman felt.

These videos were shown to the participants in the task, who were asked to rate the pain of the woman in each video on a 7-point Likert scale from 1 (“no pain at all”) to 7 (“very painful”) (see Fig. 1). Participants saw a total of 480 videos, distributed over 40 blocks with 12 trials each. Before each block, participants received two different types of information: that the person was freely showing (Label = SHOW) or suppressing (Label = SUPPRESS) the pain she felt. Unbeknownst to the participants, we included a “congruency factor,” whereby half of this information was labeled correctly, or congruently (e.g. participants were told it was a SHOW block and the woman was actually showing her pain), while the other half was labeled incorrectly, or incongruent (e.g. participants were told it was a SHOW block, but the woman was actually suppressing her pain). This division led to 10 blocks for each of the four conditions (Label-Video could either be congruent: SHOW–SHOW or SUPPRESS–SUPPRESS; or incongruent: SHOW–SUPPRESS or SUPPRESS–SHOW). The videos varied regarding the pain intensity delivered to the actress and accompanying facial reaction to the stimulation. The task was implemented in Presentation (Neurobehavioral Systems) and participants provided their answers via the keyboard.

The rTPJ was targeted using coordinate-guided neuronavigation. The coil position was identified on each participant’s scalp using the SofTaxic Navigator System (Electro Medical Systems) and the Brainsight TMS software from Rogue Research. Skull landmarks (nasion, inion, and two preauricularis points) and five points providing a uniform representation of the scalp were digitized by means of a Polaris Vicra digitizer (Northern Digital). Half of all blocks were accompanied by rTMS over the rTPJ, with the coil held by one experimenter at a 90° angle from the ear but slightly shifted between subjects depending on the live target hit shown in Neuronavigation throughout the task. For the sham condition, the coil was positioned over the vertex. Importantly, to avoid expectation-related differences between active and sham conditions, participants were told a cover story that we were interested in two brain regions, which would be stimulated during the task in separate blocks (one region on top of the head and another on the side of the head). They were also informed before that the region on the side would be more unpleasant due to its closeness to the face. In reality, only the rTPJ received active stimulation, while sham stimulation was given over the vertex, with the coil turned another 90°, so the participants experienced sounds and slight physical sensations but no actual magnetic current. The sham stimulation condition was used as a baseline to compare the effects of the active stimulation and congruency manipulation and as a control for possible placebo effects.

The two experimenters alternated holding the coil and switched between every few blocks due to the long task duration to avoid fatigue. Block order was pseudorandomized into two different orders and counterbalanced across participants (6 vs. 8 participants for each order). There were no main effects of or interactions with condition order on either the pain ratings or the RTs (all *P*’s >.511; see Table S2 in the Supplementary material). To stay in line with previous work and standards for virtual lesion studies (Paracampo et al. 2017, 2018), and to cover the whole period of emotion expression by the actress, each trial of the task included a 2s, time-locked single train of subthreshold 6 Hz stimulation (12 pulses) starting with the onset of the video, after which participants were asked to rate the pain of the woman. We chose a longer inter-stimulus-interval of >7 s (indefinite rating phase plus 6 s ITI plus a 1-s warning screen before the next stimulation) to avoid cumulative or carry-over effects of the stimulation between trials regarding (i) cognitive [see previous work, e.g. Paracampo et al. (2017) and Gallo et al. (2018)] and (ii) safety aspects [see also guidelines from previous work regarding participant safety during TMS, e.g. Chen et al. (1997) and Rossi et al. (2009)]. For an additional analysis of interactions between the TMS and trials over time, see the Supplementary material. In sum, participants received 5760 pulses if they completed all blocks. Unfortunately, three participants missed the last three blocks, and for three, we had to lower the intensity of the stimulation during the task due to unpleasantness of the stimulation. However, due to the already small sample size, we included all participants into the analysis for who we were able to calculate means for all conditions.

### Data analysis

Data analysis was conducted in RStudio (version 2021.09.0+351) and JASP (version 0.16.3.0). The two dependent variables were pain rating (i.e. the score that participants gave to the painful stimulations seen in the videos) and RT (i.e. the time it took participants to give the score expressed in milliseconds). The 12 ratings for each of the 40 blocks (total number of ratings per participant = 480) were averaged to 1 rating per block, resulting in 40 ratings and RTs per participant (with the exception of 3 participants who had 14, 37, and 37, respectively; the participant with 14 ratings was excluded from further analyses as they did not have ratings for 4/8 conditions). These 40 average ratings divided into the 3 factors with 2 levels each (Label: show vs. suppress, Video: show vs. suppress, TMS: active vs. sham), resulting in 8 block types, each shown 5 times. According to the Shapiro–Wilk test of normality, the pain ratings were normally distributed (*W* = 0.98, *P *= .25), while the RTs were not (*W* = 0.51, *P* < .001) and the presence of two important outliers was observed in a box plot of the RT data distribution. We therefore log-transformed the RT data with the log() function of R (natural logarithm), which is a common approach in research to lessen the impact of outliers or skew (Whelan 2008). The logarithmic transformation did slightly improve the data with a skewness value of −0.58 compared to 5.8 before the transformation and a kurtosis value of −0.5 compared to 43.6, suggesting that the logarithmic transformation made the distribution of RTs more symmetrical and less sharp than the original distribution. After the transformation, the previously observed outliers were no longer marked as outliers (see boxplots in the Supplementary material). Specifically, the outliers held in RTs without logarithmic transformation reported values of 43 294.25 ms and 82 671.33 ms out of an RTs average of 10 916.52 ms. After logarithmic transformation, these values were transformed to 8.97 and 9.65, respectively, out of a mean of 9.09. All RT analyses were done using the log-transformed data (but see Table S1 in the Supplementary material for a sensitivity analysis of the raw data, which did not confirm the log-transformed results). It is noteworthy that our data met the sphericity assumption, since we only have two levels of the repeated-measures factor (Hinton et al. 2004), and research shows evidence of the robustness of the repeated-measures analysis of variance (ANOVA)s to non-normality when the sphericity assumption is met (Blanca et al. 2023).

In RStudio, both dependent variables were aggregated separately, thus creating an average pain rating and an average RT for each participant for each of the eight factor combinations. In JASP, we calculated one three-way repeated-measures 2 × 2 × 2 ANOVA and one Bayesian ANOVA for each of the two dependent variables (with the three factors Label, Video, and TMS). The decision to integrate Bayesian analysis alongside frequentist methods was driven by the recognition of the complementary strengths of these approaches. While frequentist statistics offer established and widely accepted inferential tools, Bayesian methods provide a framework for incorporating prior knowledge, handling small sample sizes more effectively, providing direct relative evidence for null vs. alternative hypotheses, and delivering probability estimates directly, hopefully enhancing the robustness of findings. The interactions that emerged from the repeated-measures ANOVAs were further investigated through *post hoc* tests.

We also analyzed the correlation between the actress’s and the participants’ pain ratings. First, we calculated separate correlations for show and suppress to gauge the overall association between participants’ and actress’s ratings on a trial-by-trial basis. Then, we calculated separate correlations for each of the factors (2 TMS conditions × 2 Videos × 2 Labels), resulting in eight correlations per participant. These correlations were then fed into an ANOVA to compare differences in the ratings correlations relating to the three factors (see Table S3 and Figs S2–4 in the Supplementary material).

Due to the small sample size of the present study, not finding a significant effect could either be due to the absence of an effect, or to an effect size two small to be detected in such a small sample. To quantify how much more likely the data are under common nonzero effect sizes vs. the null effect, we also ran, as mentioned above, Bayesian analyses that provide the level of evidence for the lack of an effect, with Bayes factor (BF_incl_) < ⅓ considered to provide evidence for the lack of an effect, while ⅓ < BF_incl_ < 3 suggests a lack of power to adjudicate whether there is a (weak) effect or not (see Keysers et al. 2020).

## Results

### Pain ratings

**Figure 2.**
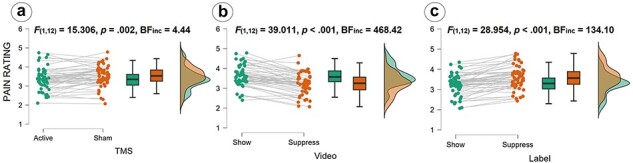
Significant pain rating results show (a) a main effect of TMS, (b) a main effect of Video: and (c) a main effect of Label; Please note that videos in which the actress had suppressed her pain were sometimes labeled as “show” and sometimes as “suppress”, with the same videos being shown for the factor label; The scattered dots in the graph on the left represent the averaged ratings given by the participants (eight per participant, one for each of the eight combinations between factors levels); Lines between dots represent ratings of the same participants; The boxplots describe the distribution of ratings, the dark line in the middle of the box being the median, the top/bottom of the box being the 75^th^/25^th^ percentile, respectively, and the top/bottom of the whisker being the maximum/minimum; The density plots show the distribution of the ratings with the peaks being the points where most of the values are concentrated.

We observed a main effect of TMS (*F*_(1,12)_ = 15.30, *P *= .002, *η*^2^ = 0.04; see Fig. 2a and Table 1). The corresponding Bayesian ANOVA provided moderate evidence for H1 (BF_incl_ = 4.44). Participants rated the pain of the actress higher in the sham TMS (*M* = 3.51, SE = 0.12) than in the active TMS condition (*M* = 3.34, SE = 0.12).

**Table 1.** Repeated-measures ANOVA of the pain ratings.

Effects	*F*(1,12)	*P*	*η* ^2^	BF_incl_
**TMS**	**15.306**	**.002**	**0.047**	**4.446**
**Label**	**28.954**	**<.001**	**0.213**	**134.104**
**Video**	**39.011**	**<.001**	**0.272**	**468.422**
TMS × Label	0.021	.886	<0.001	0.742
TMS × Video	0.055	.818	<0.001	0.874
Label × Video	2.355	.151	0.007	1.377
TMS × Label × Video	0.366	.557	0.002	**0.247**

Notes: Type III sum of squares. Significant effects are marked in bold. The last column indicates the result of a Bayesian ANOVA on the same data; BF >3 indicates evidence for an effect; BF<0.33 indicates evidence of absence of an effect.

Moreover, we observed expected main effects of Video (*F*_(1,12)_ = 39.01, *P *< .001, *η^2^ *= 0.27; see Fig. 2b) and Label (*F*_(1,12)_ = 28.95, *P *< .001, *η^2^ *= 0.21; see Fig. 2c), which were strongly confirmed by the Bayesian analysis (Video: BF_incl_ = 468.42; Label: BF_incl_ = 134.10). The main effect of Video showed that the participants gave higher ratings for videos in which the actress was told to express her emotions freely (“show” condition; *M* = 3.62, SE = 0.12) than for videos in which she was told to suppress her emotions (“suppress” condition; *M* = 3.22, SE = 0.11). In contrast, the main effect of Label showed that the participants gave higher ratings if they had been previously told that the actress had been instructed to suppress her emotions (“suppress” condition; *M* = 3.60, SE = 0.12) than when they had been previously told that the actress had been instructed to express her emotions freely (“show” condition; *M* = 3.24, SE = 0.12).

No interaction effects were found between Label × Video (*F*_(1,12)_ = 2.35, *P *= .151, *η^2^ *< 0.01), TMS × Video (*F*_(1,12)_ = 0.05, *P *= .818, *η^2^ *< 0.01), TMS × Label (*F*_(1,12)_ = 0.02, *P *= .886, *η^2^ *< 0.01), and TMS × Label × Video (*F*_(1,12)_ = 0.36, *P *= .55, *η^2^ *< 0.01). The lack of any interaction effects was partially confirmed by the Bayesian ANOVA (Label × Video: BF_incl_ = 1.37; TMS × Video: BF_incl_ = 0.87; TMS × Label: BF_incl_ = 0.74; TMS × Label × Video: BF_incl_ = 0.24), although many effects had inconclusive Bayes Factors between 3 and 0.33 with little evidence for either H1 or H0 (see Table 2).

**Table 2.** Repeated-measures ANOVA of the RTs for the pain ratings.

Effects	*F* (1,12)	*P*	*η* ^2^	BF_incl_
TMS	1.095	.316	0.004	**9.28**
Label	4.129	.065^T^	0.013	**5.192**
TMS × Label	1.438	.254	<0.001	1.548
Video	0.156	.700	0.007	**19.366**
**TMS × Video**	**21.207**	**<.001**	**0.321**	**35.953**
**Label × Video**	**14.413**	**.003**	**0.095**	**16.360**
TMS × Label × Video	0.588	.458	0.006	**3.531**

Notes: Type III sum of squares. Significant effects are marked in bold. The last column indicates the result of a Bayesian ANOVA on the same data; BF >3 indicates evidence for an effect; BF < 0.33 indicates evidence of absence of an effect.

### Response times

**Figure 3.**
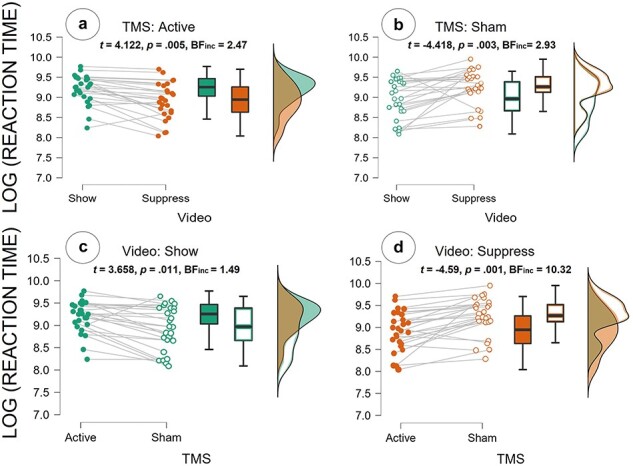
The reaction time results show an interaction effect of TMS × Video: (a) comparison between TMS “Active” (full dots) with Video “show” and Video “suppress”: (b) Comparison between TMS “Sham” (empty dots) with Video “show” and Video “suppress”; (c) Comparison between Video “show” with TMS “active” (full dots) and TMS “sham” (empty dots); (d) Comparison between Video “suppress” with TMS “active” (full dots) and TMS “sham” (empty dots); The scattered dots in the graph on the left represent the averaged ratings given by the participants (8 per participant, 1 for each of the 8 combinations between factors levels); Lines between dots represent ratings of the same participants; The boxplots describe the distribution of ratings, dark line in the middle of the box being the median, the top/bottom of the box being the 75^th^/25^th^ percentile, respectively, and the top/bottom of the whisker being the maximum/minimum; The density plots show the distribution of the ratings with the peaks being the points where most of the values are concentrated.

We observed a significant interaction effect between TMS and Video (*F*_(1,12)_ = 21.207; *P* < .001, *η^2^ *= 0.32; see Fig. 3a–d and Tables 2 and 3), showing that rTMS had a different effect on the RTs in the show vs. suppress videos. Bayesian analysis provided additional strong evidence for this interaction (BF_incl_ = 35.95), and Bonferroni-corrected (for a family of six estimates) *post hoc* analysis revealed that under the sham TMS condition, participants were faster to give ratings to videos in which the actress was freely showing her emotions (*M *= 8.93, SE = 0.11) than when she was suppressing it (*M *= 9.24, SE = 0.11; see Fig. 3a). This effect was altered during active 6 Hz rTPJ stimulation, whereby participants were faster to give ratings in the “suppress” condition (*M* = 8.91, SE = 0.11) compared to the “show” condition (*M* = 9.20, SE = 0.11; see Fig. 3b). Furthermore, participants were faster to rate freely expressed pain under sham (*M* = 8.93, SE = 0.11) compared to active perturbation of the rTPJ (*M* = 9.20, SE = 0.11; see Fig. 3c). In contrast, they were faster to rate suppressed pain under active (*M* = 8.91, SE = 0.11) compared to sham stimulation (*M* = 9.24, SE = 0.1; see Fig. 3d).

**Table 3.** *Post hoc* comparison for TMS × Video.

Comparisons	Mean difference		SE	*t*	*P*_bonf_	Cohen’s *d*	BF_incl_
**Active show**	**Sham show^c^**	**0.266**	**0.073**	**3.658**	**.011**	**0.612**	1.49
	**Active Suppress^a^**	**0.289**	**0.070**	**4.122**	**.005**	**0.666**	2.47
	Sham suppress	−0.044	0.042	−1.061	>.999	−0.102	**0.25**
**Sham show**	Active suppress	0.023	0.042	0.563	>.999	0.054	**0.22**
	**Sham suppress^b^**	**−0.310**	**0.070**	**−4.418**	**.003**	**−0.713**	2.93
**Active suppress**	**Sham suppress^d^**	**−0.333**	**0.073**	**−4.59**	**.001**	**−0.767**	**10.32**

Notes: Significant effects are marked in bold. The last column indicates the result of Bayesian *t*-tests on the same data; BF >3 indicates evidence for an effect; BF < 0.33 indicates evidence of absence of an effect. ^a, b, c, d^Denote the effects given in Fig. 3.

**Figure 4.**
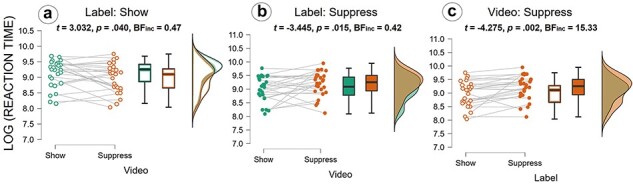
The reaction time results show an interaction effect of Label × Video: (a) comparison between Label “show” (empty dots) with Video “show” and Video “suppress”; (b) Comparison between Label “suppress” (full dots) with Video “show” and Video “suppress”; (c) Comparison between Video “suppress” with Label “show” (empty dots) and Label “suppress” (full dots); The scattered dots in the graph on the left represent the averaged ratings given by the participants (8 per participant, 1 for each of the 8 combinations between factors levels); Lines between dots represent ratings of the same participants; The boxplots describe the distribution of ratings, dark line in the middle of the box being the median, the top/bottom of the box being the 75^th^/25^th^ percentile, respectively, and the top/bottom of the whisker being the maximum/minimum; The density plots show the distribution of the ratings with the peaks being the points where most of the values are concentrated.

We also found an interaction between Label and Video (*F*_(1,12)_ = 14.413; *P* = .003, *η^2^* =0.09; see Fig. 4a–c and Tables 2 and 4), showing that the RTs during videos labeled as show vs. suppress were influenced differently by the label. In line with this, the effect was also strongly confirmed by the Bayesian analysis (BF_incl_ = 16.36). *Post hoc* analysis showed that when participants were previously told that the actress would freely show her pain, they were faster to give ratings for the videos in which the actress actually suppressed the pain (*M *= 8.96, SE = 0.12) vs. when she actually showed it (*M *= 9.12, SE = 0.10; see Fig. 4a). On the other hand, when participants were previously told that the actress would suppress the pain, they were faster to give ratings for the videos in which the actress actually showed her pain (*M* = 9.02, SE = 0.12) vs. when she actually suppressed it (*M* = 9.19, SE = 0.09; see Fig. 4b). *Post hoc* tests further showed that in the videos with suppressed pain, the Label “show” produced faster RTs (*M* = 8.96, SE = 0.12) compared to Label “suppress” (*M* = 9.19, SE = 0.09; see Fig. 3d). Note that in the videos with freely showing pain, there was no significant difference in RTs between the two Labels (“show”: *M* = 9.12, SE = 0.10; “suppress”: *M* = 9.02, SE = 0.12).

**Table 4.** *Post hoc* comparison for Label × Video.

Comparisons	Mean difference		SE	*t*	*P*_bonf_	Cohen’s *d*	BF_incl_
**Show show**	Suppress show	0.102	0.052	1.943	.391	0.235	0.76
	**Show suppress^a^**	**0.153**	**0.050**	**3.032**	**.040**	**0.351**	0.47
	Suppress suppress	−0.072	0.040	−1.790	.517	−0.165	**0.24**
**Suppress show**	Show suppress	0.051	0.040	1.271	>.999	0.117	**0.23**
	**Suppress suppress^b^**	**−0.173**	**0.050**	**−3.445**	**.015**	**−0.399**	0.42
**Show suppress**	**Suppress suppress^c^**	**−0.224**	**0.052**	**−4.275**	**.002**	**−0.516**	**15.33**

Notes: Significant effects are marked in bold. The last column indicates the result of Bayesian *t*-tests on the same data; BF >3 indicates evidence for an effect; BF < 0.33 indicates evidence of absence of an effect. ^a, b, c, d^Denote the effects given in Fig. 3.

We found no main effects of TMS (*F*_(1,12)_ = 1.09, *P *= .316, *η^2^ *< 0.01) or Video (*F*_(1,12)_ = 0.15, *P *= .700, *η^2^ *< 0.01), while a trend emerged for Label (*F*_(1,12)_ = 4.12, *P *= .065, *η^2^ *= 0.01). The Bayesian ANOVA provided moderate evidence for the Label effect (BF_incl_ = 5.19), which would suggest a faster rating when being told that the actress will freely show (*M* = 9.04, SE = 0.10) vs. suppress her pain (*M* = 9.10, SE = 0.10). Finally, no interaction effects were observed between TMS × Label (*F*_(1,12)_ = 1.43, *P *= .254, *η^2^ *< 0.01) and TMS × Video × Label (*F*_(1,12)_ = 0.58, *P *= .458, *η^2^ *< 0.01). However, in contrast to the frequentist ANOVA, the Bayesian ANOVA suggested strong-to-moderate evidence for both the inclusion of the main effects of TMS (BF_incl_ = 9.28) and Video (BF_incl_ = 19.36) and the interaction between TMS × Video × Label (BF_incl_ = 3.53). It is noteworthy that for both significant interaction effects (TMS × Video; Label × Video), pairwise Bayesian comparison showed evidence of an effect only in Fig. 3d and Fig. 4c.

Importantly, TMS did not alter the accuracy of the ratings (we quantified accuracy as the correlation between the trial-by-trial ratings of the participant and those of the actress herself; main effect of TMS, *F*(1,12) = 2.09, *P* = .174, BF_incl_ = 0.42; see Supplementary material).

## Discussion

This study investigated the rTPJ’s causal contribution to pain empathy when people are given contextual information about others’ intentions to freely display or suppress their pain. Our findings showed that TMS on the rTPJ influenced both participants’ empathic perception and the speed at which they provided ratings.

Active 6 Hz rTPJ stimulation lowered participants’ empathic pain ratings. This result confirms previous work emphasizing the importance of the rTPJ in the processing of social stimuli (Decety and Lamm 2007, Carter and Huettel 2013). In line with our results, rTPJ perturbation reduced the intensity of pain perceived in others (Coll et al. 2017, Miller et al. 2020), which confirms that rTPJ inhibition may reduce behavioral and brain measures related to the cognitive–evaluative component of empathy.

Behavioral results of the rating data also showed that participants gave higher pain intensity ratings to observed pain that was openly shown in the videos, compared to suppressed pain, regardless of the contextual information provided beforehand. This result is in line with studies where people rate freely expressed pain higher than suppressed pain (Poole and Craig 1992).

Additionally, results revealed that people overestimated the pain of the actress when they were told that she was suppressing it, regardless of the actual video. This is consistent with studies showing that contextual information modulates the perception and recognition of facial emotions (Milanak and Berenbaum 2014), especially when they are more difficult to decode (Bublatzky et al. 2020). It has been suggested that when the inferential weight is higher and multiple information sources are present, in our case the video and the label, the observer may privilege one of the two information sources, making one more salient than the other (Mendolia 2021). Specifically, the fact that the “suppress” label significantly increased the perceived pain could depend on an overcompensation mechanism adopted by the participants when faced with emotions that are more difficult to decode. Not only does the suppressed pain expression in itself require more inferential effort, but also the previous information indicating that pain will be suppressed creates the expectation of such inferential effort. Klein (2019) suggested that overestimating others’ emotions may also be a mechanism for social approval motives, i.e. people prefer to overestimate others’ emotions because this indicates their effort and empathy.

Of note, we would have expected a modulation effect of TMS by the given contextual information, but it should be mentioned that the contextual information used here did not completely overlap with the information used in previous studies investigating the role of the TPJ in modulating the social framing effect (Gu et al. 2019). The specific nature of the contextual information we used might explain the lack of any interactive effect with rTPJ stimulation. Specifically, the contextual information might have been perceived by participants as competing with the information presented in the video, especially under conditions of incongruence. Other studies showed that the TPJ was specifically involved in mental state attribution but not in executive functions, such as response selection among competitors, suggesting that the TPJ might be less involved in processes of detection and resolution of incongruities (Vistoli et al. 2016).

Contrary to what we expected, no interaction effect between Label and Video emerged from the frequentist ANOVA, although the Bayesian ANOVA did not provide conclusive evidence.

Regarding participants’ RTs, evidence of an interactive effect between TMS and Video emerged. Under sham stimulation, participants were quicker to make judgments about the pain experienced by the person in the video when it was openly shown rather than suppressed, regardless of the information previously provided. Analogous to the pain rating results, this finding could intuitively be explained by the fact that inferring someone else’s pain when it is suppressed may require more cognitive effort compared to situations in which pain is openly shown. This enhanced cognitive demand could plausibly translate into longer RTs. Importantly, this effect did not seem to be affected by potentially cumulative TMS over time (see exploratory analysis in the Supplementary material).

Interestingly, under active stimulation, the previous result was reversed: when the rTPJ was disrupted, subjects were faster to give ratings when pain was suppressed rather than openly shown. This result supports the role of the rTPJ in the speed of social judgment (Costa et al. 2008). More specifically, *post hoc* tests revealed a slowing of RTs for the “show” condition and a speeding up in the “suppress” condition under active TMS. The former aligns well with the main effect of TMS on ratings: since the rTPJ is recruited in the process of pain attribution, its perturbation could slow down the speed of inference as well as the perceived intensity of pain (Coll et al. 2017). The speeded RTs in the “suppress” condition, which was confirmed with strong evidence by Bayesian *post hoc* comparison, are an interesting findings worth discussing. Assuming that the level of inferential demand may differ between the “show” and “suppress” conditions, such that suppress stimuli may require more effort to make inferences about the pain of others, it is possible to hypothesize that the reversal of RTs between the sham and active TMS depends on the involvement of the rTPJ in supporting such complex inferences. Specifically, if the TPJ supports difficult inferences about others’ mental states, which could result in longer RTs due to increased pondering, its disruption might compromise this pondering process by making the subjects’ judgment more reckless, resulting in shorter RTs. This hypothesis is supported by studies that have indeed shown stronger activation in the rTPJ as inferential demands increased (Vistoli et al. 2016, Henry et al. 2021). Moreover, the contextual updating hypothesis (Geng and Vossel 2013) suggests that the TPJ is essential for the evaluation and integration of stimulus information with internal models of task performance and expectations. It would appear that mismatches between new sensory information and expectations produce the greatest responses from the TPJ because they represent the most significant updates to the internal model. It is possible that suppress videos represent a form of violation of the default assumption that a person in pain should show facial expressions of pain. Such a violation should engage the rTPJ more to update the internal model, and this increased engagement could result in longer decision times during pain evaluation. Disruption of the rTPJ could break this updating mechanism and the associated commitment, shortening the subjects’ RTs.

Contrary to what we expected, we found evidence of the absence for a modulation effect of TMS on RTs regarding contextual information. This result is in line with our rating data and suggests that the rTPJ region we targeted is less involved in using contextual information for social judgments.

Regarding the interactive effect between the label and the video, it appears that participants who thought the actress showed pain were quicker raters when a video was presented where the actress suppressed the pain; conversely, when they thought the actress suppressed pain, they were quicker with videos where the actress openly showed pain. These results suggest that in the presence of information about pain suppression, whether presented in the video or suggested in the label, participants relied on the simpler source to evaluate, either the label “show” (in the case of concurrent video “suppress”) or the video “show” (in the case of concurrent label “suppress”). This confirms the hypothesis that as the inferential weight increases, there is a tendency to focus on a single source of information (Mendolia 2021). This would explain why participants’ RTs were slower in the condition of concurrent video “suppress” and label “suppress”: it is possible that the source that became salient in the decision-making process was the “show” label, i.e. the easiest to process. However, it should be noted that it remains counterintuitive that participants were not significantly faster in the condition of concurrent video “show” and label “show.” Therefore, further studies investigating the relationship between videos and contextual information are necessary.

The present study has some strengths and limitations worth mentioning. First, a small sample size, resulting in lower power, may have hidden smaller effects. The present study thus needs replication, especially for the effects with inconclusive BFs. Second, given the task length and unpleasantness of rTMS, some subjects did not complete the entire task. Relatedly, we unfortunately did not collect ratings of first-hand pain or unpleasantness of the TMS. It is reasonable to assume that active TMS over the TPJ produced more discomfort than applying TMS away from the head over the vertex. This discomfort could have interfered with participants’ empathic responses, e.g. via shared representations and/or vicarious pain experiences (Keysers et al. 2010, Lamm et al. 2016). Indeed, previous research suggests that subjective discomfort was strongly correlated with changes in reaction time, particularly for stimulation of parietal regions (Holmes and Meteyard 2018). Although the stimulation was the same between conditions and likely affected all conditions equally, and participants had indefinite time to answer, leaving them plenty of time after the stimulation to make their judgment, it cannot be fully ruled out that our outcome variables were influenced by the discomfort caused by the active TMS condition vs. the sham condition. Our exploratory analysis of the interaction between the TMS and effects over time indicates that this explanation is unlikely for the RTs but could have been a potential confounder for the pain ratings (see Supplementary material). Whether this interaction for the latter was due to the cumulative TMS effect or an increasing unpleasantness of the stimulation over time will have to be systematically investigated in future work. Third, we did not measure self-experience ratings. Many empathy models propose that incorporating one’s own affective experiences, such as felt unpleasantness when perceiving others in pain, is crucial for comprehending the experiences of others. This should be considered in future studies. Fourth, due to limited resources, we were not able to collect participant-specific functional brain images and thus individual coordinates of the rTPJ. Instead we opted for stimulating the same coordinate, derived from previous literature, in all participants. Note that this region therefore likely differs between individuals. The lack of individualized localization of the TPJ may have diminished our success in accurately identifying the region in all participants. An alternative, potentially less noisy approach would have been to run a functional MRI localizer for each participant, in which participants would perform a series of tasks, possibly including the task presented in this manuscript, shown to reliably recruit the TPJ. From this localizer, we could then have estimated TPJ coordinates specific to each participant. Fifth, since the participants had indefinite time to provide their answers, we measured the response time instead of reaction time. This has to be considered when interpreting the present findings as faster responses might be related to more spontaneous judgments. Last, it should be acknowledged that the empathic response to pain may be modulated by the traits and characteristics of the target (De Vignemont and Singer 2006, Aue et al. 2021). Given the presence of only one target individual (e.g. female, Caucasian, and young) in our videos, it is possible that our results may not be readily generalizable to other targets. Future studies could replicate by adding a broader range of target individuals with different phenotypic traits among them. Despite these limitations, the present study partly confirms and expands the previous literature, while providing new evidence of the rTPJ’s role in judging others’ pain, aligning with the perspective that, in some situations, mentalizing processes are engaged together with simulative processes to infer others’ pain. Our results are underlined regarding their robustness through concurrent Frequentist and Bayesian analyses, as well as Bonferroni-corrected *post hoc* effects.

In conclusion, this study provides new insights into the involvement of the rTPJ in inferences about others’ pain. If these findings are replicated, they may indicate that the rTPJ can be considered a valid target for stimulation when studying the mechanisms of self–other control in socioemotional processes such as empathy. Shedding light on the role of the rTPJ in the process of attributing pain to others should be considered important in order to gain a deeper understanding of both the contribution of this brain region to social judgment and the phenomenon of empathy for pain. Furthermore, comprehension of the rTPJ’s involvement in empathy for pain could provide useful clinical tools for dealing with conditions in which this ability is impaired.

## Supplementary Material

nsae057_Supp

## Data Availability

The raw and aggregated datasets for this study can be found in the Supplementary material.
