# Supplementary material for: Cognitive control: exploring the causal role of the rTPJ in empathy for pain mediated by contextual information
Source: Soc Cogn Affect Neurosci. 2024 Sep 6;19(1):nsae057. doi: 10.1093/scan/nsae057 (PMC11414476; doi:10.1093/scan/nsae057)
Supplement: nsae057_Supp [file nsae057_supp.zip › scan-23-258-File010.pdf]

Supplement to

Cognitive Control: Exploring the causal role of the rTPJ in empathy for pain mediated by contextual information

Helena Hartmann[1,2,*+], Egle M. Orlando[1,3,+], Karina Borja[1], Christian Keysers[1,4,‡], & Valeria Gazzola[1,4,‡]

[1] *Social Brain Lab, Netherlands Institute for Neuroscience, Royal Netherlands Academy of Art and Sciences, Amsterdam, the Netherlands*

[2] *Clinical Neurosciences, Department for Neurology and Center for Translational and Behavioral Neuroscience, University Hospital Essen, Germany*

[3] *Department of General Psychology, University of Padova, Italy*

[4] *Brain and Cognition, Department of Psychology, University of Amsterdam, The Netherlands.*

* Corresponding author: Helena Hartmann, helena.hartmann@uk-essen.de, Clinical Neurosciences, Department for Neurology, University Hospital Essen, Essen, Germany

[+] These authors contributed equally and share first-authorship.

[‡] These authors contributed equally and share last authorship.

Supplementary Materials

Log-transformation of response times (RTs)

As visible in Figure S1, the log transformation made the distribution of RTs more symmetrical and less sharp than the original distribution.

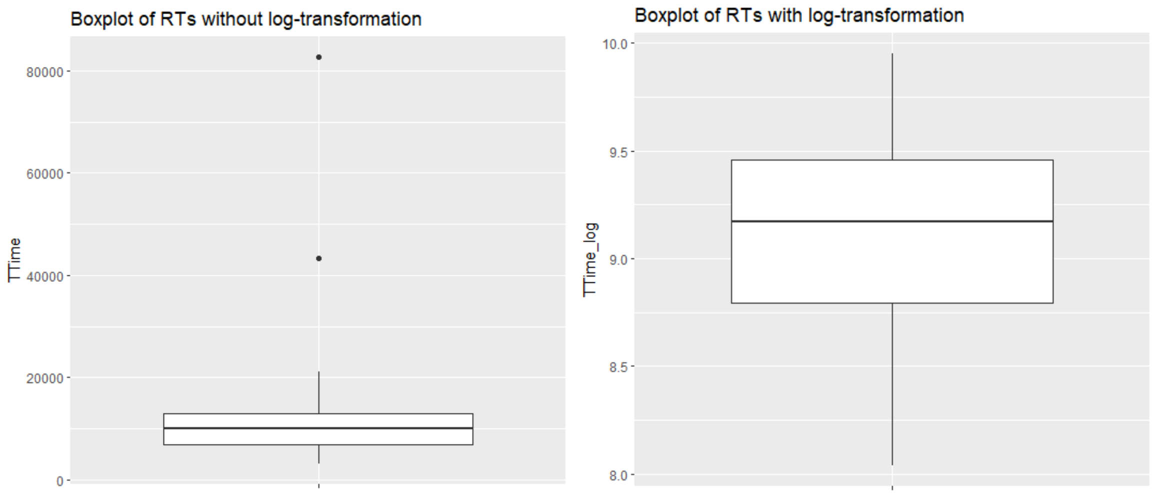

Figure S1. Box-plot visualization of response times before (left, 10[th]s of milliseconds) and after (right) log-transformation.

Analysis of untransformed response times

By performing the same analyses on the un-transformed RTs, the effects observed on the log-transformed RTs are no longer present (see Table S1). Bayesian analyses also indicate evidence of the absence of the effect.

Table S1
ANOVA of untransformed response times.

Effects	*F (1,12)*	*p*	BF_{incl}
TMS	0.436	.510	0.245
Video	0.061	.805	0.208
Label	0.533	.467	0.248
TMS x Label	0.014	.905	0.060
TMS x Video	0.225	.636	0.048
Label x Video	1.614	.207	0.050
TMS x Label x Video	2.233	.138	0.012

Inclusion of condition order as a factor

By adding the additional variable “condition order”, the statistical significance of the observed effects was not altered (Table S2). No significant effect of the order was found.

Table S2
Frequentist ANOVA on pain rating and response time, with the additional factor “order of administration of TMS condition”. Two different pseudorandomized orders were used.

	Pain Rating		*Response Time*	
Effects	*F(1,80)*	*p*	*F(1,80)*	*p*
Order	0.457	.511	0.007	.934
TMS	**5.183**	**.023**	0.896	.346
Label	**42.52**	**< .001**	2.543	.114
Video	**49.74**	**< .001**	0.013	.906
Video x Order	0.686	.409	0.110	.740
Label x Order	2.087	.152	0.387	.535
Label x Video	2.841	.095	**11.33**	**.001**
TMS x Order	0.195	.659	0.862	.355
TMS x Video	0.057	.811	**47.03**	**< .001**
TMS x Label	0.226	.635	0.698	.405
Order x Label x Video	2.686	.105	0.081	.775
Order x TMS x Video	0.007	.933	0.664	.417
Order x TMS x Label	0.419	.518	0.229	.633
TMS x Label x Video	1.051	.308	1.052	.308
TMS x Label x Video x Order	1.844	.178	0.060	.806

Correlation between participants' and actress' pain ratings

The trial-by-trial correlations of the standardized ratings between participants' empathy and actress' pain ratings revealed significant positive correlations both for the show ($r_{Pearson}$ = .70, $p < .001$, $BF_{incl} > 100$) and suppress ($r_{Pearson}$ = .47, $p < .001$, $BF_{incl} > 100$) conditions.

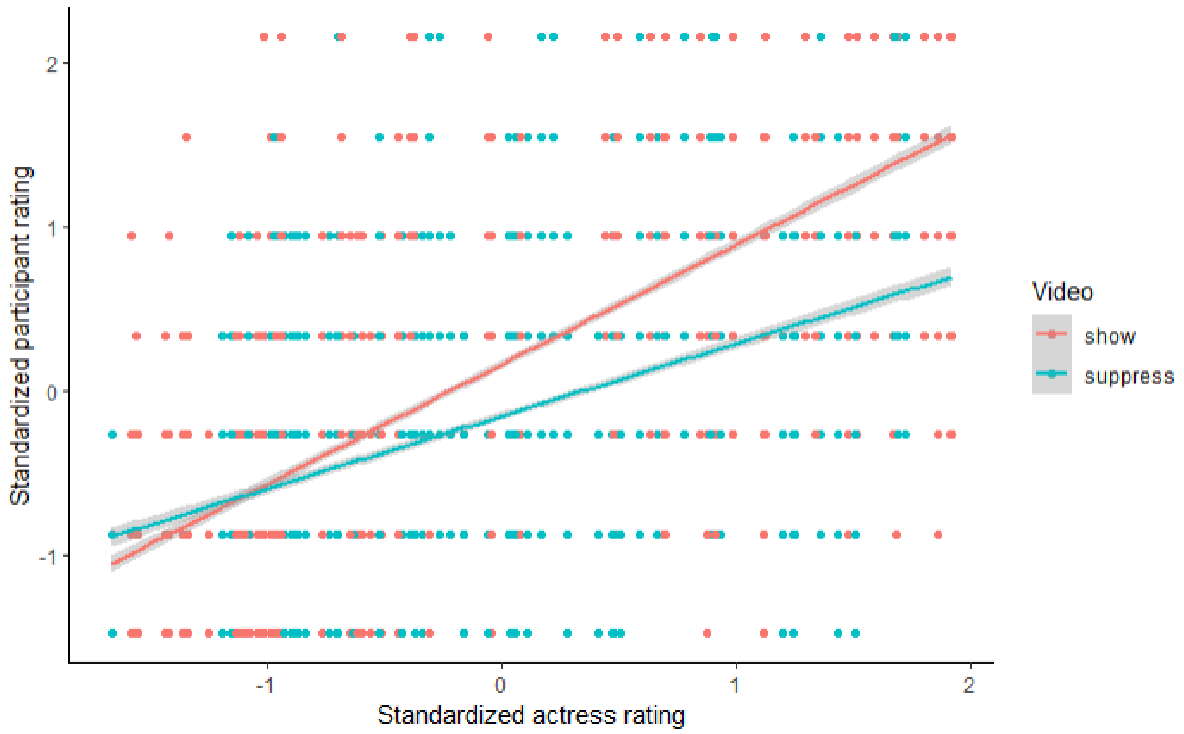

Figure S2. Significantly positive correlations between participants' empathic and actress' pain ratings during videos where the actress either suppressed vs. showed her pain (averaged over the TMS and label factors). Each dot represents one trial in either the show or suppress condition.

The separate correlations for each of the 3 factors with 2 levels each fed into an ANOVA revealed a significant main effect of video ($F(1,12) = 194.50$, $p < .001$, $BF_{incl} > 100$; see Figure S2): The correlation between the actress' and the participants' ratings was higher when the actress freely showed ($r = 0.74$) vs. suppressed her pain ($r = 0.53$; see Figure S3). No other effects reached significance (see Table S3).

Table S3
Frequentist ANOVA of correlations between participants' empathic and actress' pain ratings, separate for the three factors, and the BF_{incl} from the corresponding Bayesian ANOVA.

Effects	*F (1,12)*	*p*	BF_{incl}
TMS	2.09	.174	0.422
Video	194.50	< .001	$1.122e^{+6}$
Label	0.00	.969	0.239
TMS x Label	0.26	.619	0.177
TMS x Video	0.98	.342	0.468
Label x Video	0.21	.653	0.309
TMS x Label x Video	0.01	.910	0.046

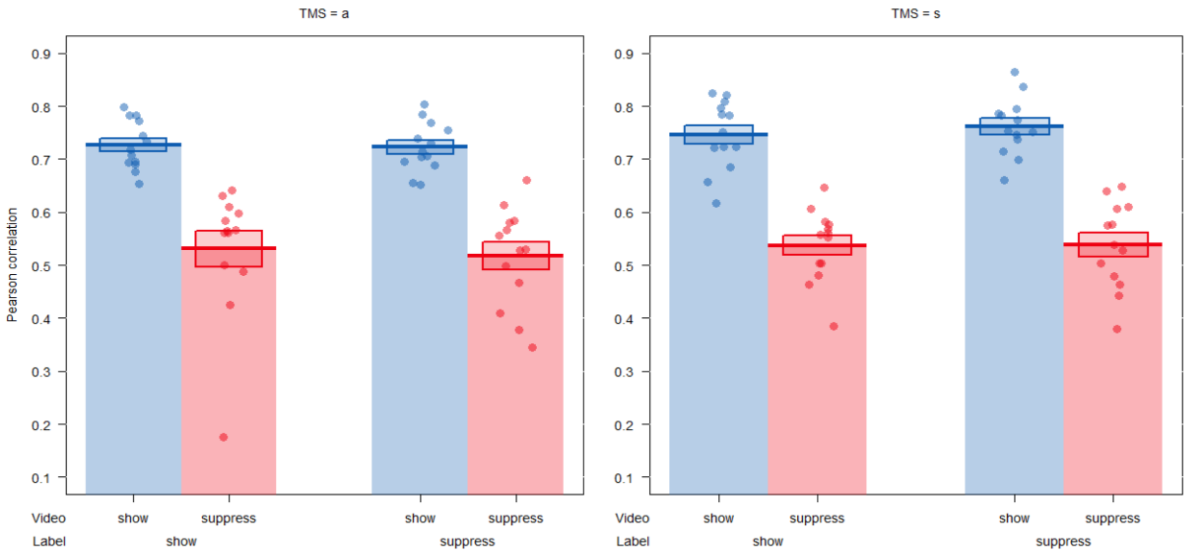

Figure S3. Significantly lower correlation between participants' empathic and actress' pain ratings during videos where the actress suppressed vs. showed her pain (main effect of video), but no other effects emerged, specifically no effect of TMS. a = active, s = sham. Each dot represents one participant in a certain condition (e.g. show - show or show - suppress), bold bars show the mean and box indicates the standard error of the mean.

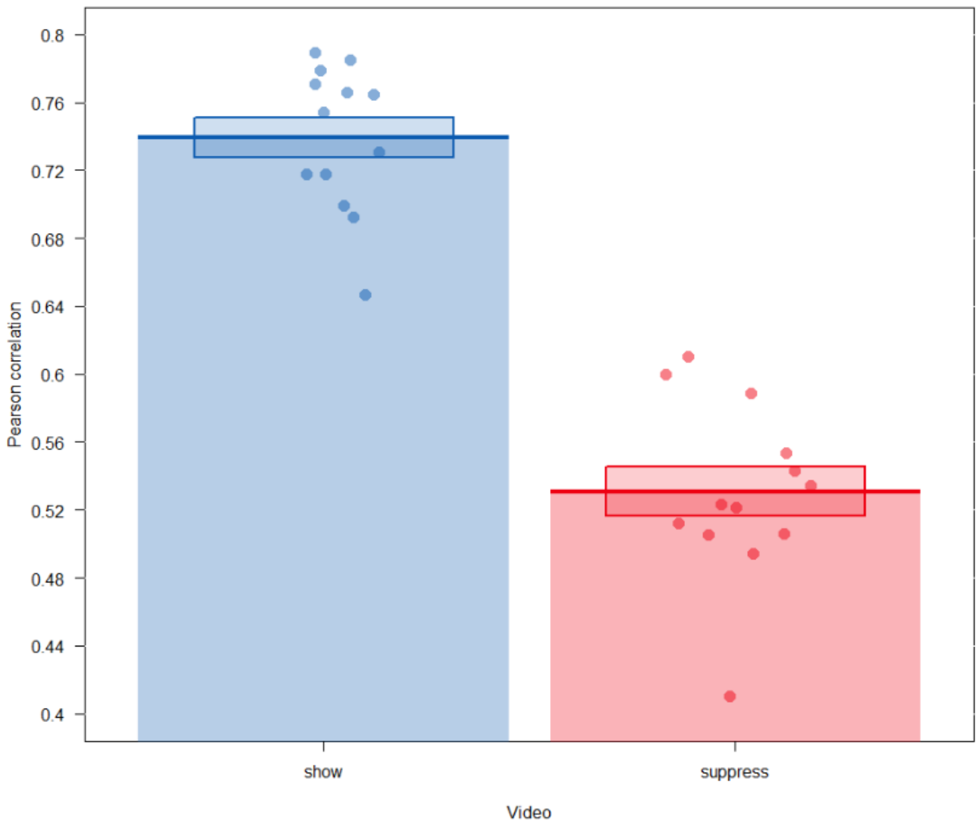

Figure S4. Significantly lower correlation between participants' empathic and actress' pain ratings during videos where the actress suppressed vs. showed her pain (main effect of video, with all other conditions averaged). Each dot represents one participant in either the show or suppress condition, bold bars show the mean and box indicates the standard error of the mean.

Interaction between TMS and trials over time

To test whether there was a cumulative TMS effect over time, we performed two ANOVAs for response times and pain ratings, this time including trial number as an explanatory (continuous) factor in addition to the factor TMS (active vs sham), while averaging over the other factors Video and Label. We reasoned that if the effect of TMS is cumulative, the difference between sham and active TMS should increase over trials, i.e. show a trial x TMS interaction.

Regarding the response times, we observed an expected significant main effect of trial number, whereby participants generally provided faster responses over time (F(1,6360.8) = 739.96, $p < .001$). Importantly, there was no interaction of trial number with TMS ($p > .949$), indicating that TMS did not have cumulative effects on the response times. The Bayesian

counterpart of this analysis also indicated the highest BF for the model including only trial number (BF_{incl} > 100).

Regarding the pain ratings, we observed a significant interaction between TMS and trial number, whereby pain ratings decreased in the active and increased in the sham condition (F(1,6355.8) = 69.99, p < .001; with the highest BF being present for the model including the interaction, BF_{incl} > 100, see Figure S5). This indicates that over time, the active TMS stimulation changed participants' empathic ratings to be lower, compared to sham.

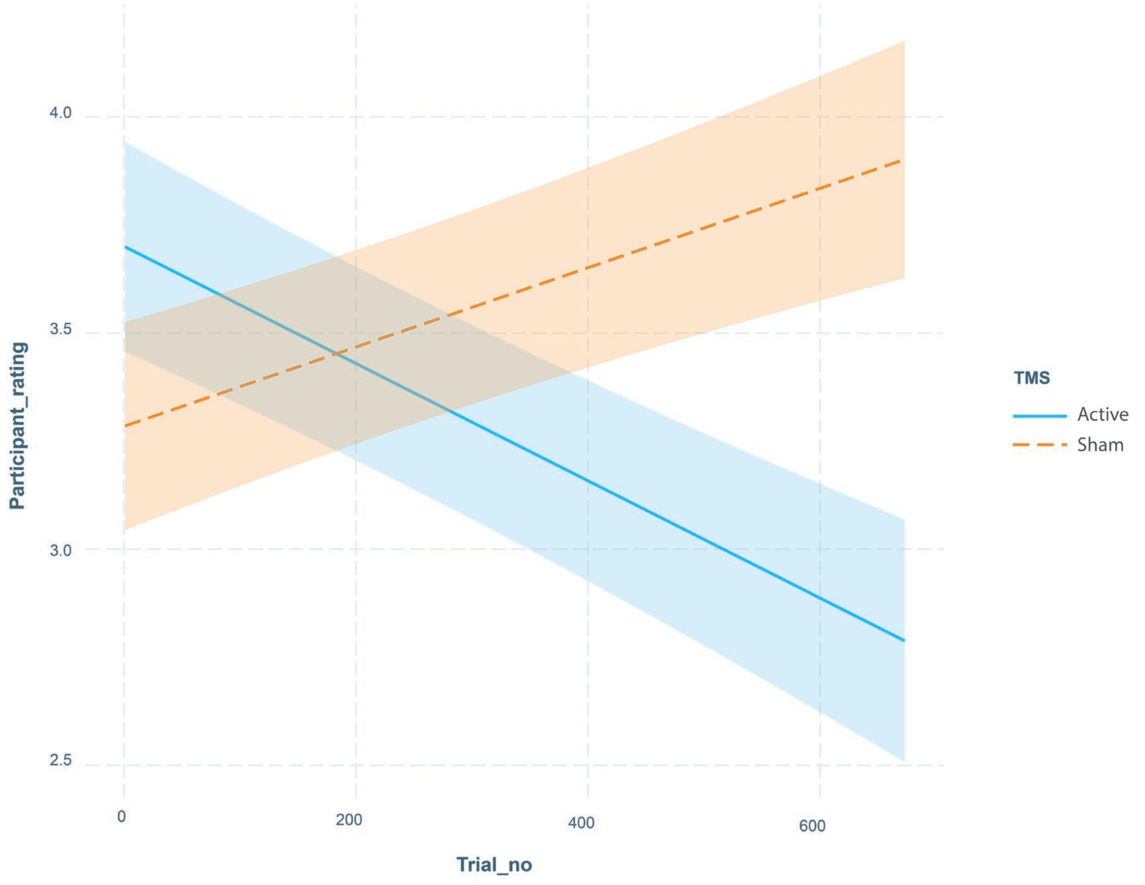

Figure S5. Significant interaction between TMS and trial number, showing that participants rated the pain of the actress lower in the active (blue) compared to the sham (orange) condition over time. The regression line is surrounded by a band showing the 95% confidence interval.
